# COMPARISON OF STEREOLOGY METHODS FOR ASSESSING AGE-RELATED EFFECTS ON IMMUNOSTAINED BRAIN CELLS

**DOI:** 10.1093/geroni/igad104.2232

**Published:** 2023-12-21

**Authors:** Grant Denham, Saeed Alahmari, Aidan Anderson, Krystal Sanchez, Dominick Dag, Lawrence Hall, Dmitry Goldgof, Peter Mouton

**Affiliations:** Stereology Resource Center Biosciences, Tampa, Florida, United States; Najran University, Najran, Najran, Saudi Arabia; Stereology Resource Center Biosciences, Tampa, Florida, United States; Stereology Resource Center Biosciences, Tampa, Florida, United States; Stereology Resource Center Biosciences, Tampa, Florida, United States; University of South Florida, Tampa, Florida, United States; University of South Florida, Tampa, Florida, United States; Stereology Resource Center Biosciences, Tampa, Florida, United States

## Abstract

The primary benefit of stereology methods is quantification of well-stained biological objects in tissue sections with the ability to adjust sampling intensity to achieve desired levels of precision. The advent of hand-crafted algorithms and artificial intelligence-based deep learning (DL) provides an opportunity for more standardized collection of stereology data with enhanced efficiency and higher reproducibility compared to state-of-the-art manual stereology. We contrasted and compared the performance of four manual, semi-automatic, and fully automatic approaches for generating data for total number of Neu-N immunostained neurons in neocortex (NCTX) in the mouse brain. The gold standard for these studies was manual counts using the state-of-the-art optical fractionator method on 3-D reconstructed serial z-axis image stacks through a known tissue volume (disector stacks). To allow for direct methodological comparisons on the same images, disector stacks were automatically converted into extended depth of field (EDF) images in which all neurons in the disector stack were imaged at each cell’s maximal plane of focus. Total number of Neu-N neurons on the same EDF images were counted by a fully automatic hand-crafted method [automatic segmentation algorithm (ASA)] and a semi-automatic method [ASA counts manually corrected for false positives and negatives]. All comparison counts were done using unbiased frames and counting rules with total counts of NeuN-immunostained neurons by the optical fractionator method. The results were comparable across methods with wide variations in throughput efficiency and inter-rater agreement. These results are discussed with respect to applications to experimental studies of brain aging, neuroinflammation and neurodegenerative disease.

