# Correction to: COMPARISON OF STEREOLOGY METHODS FOR ASSESSING AGE-RELATED EFFECTS ON IMMUNOSTAINED BRAIN CELLS

**DOI:** 10.1093/geroni/igae027

**Published:** 2024-03-06

This is a correction to: Grant Denham, Saeed Alahmari, Aidan Anderson, Krystal Sanchez, Dominick Dag, Lawrence Hall, Dmitry Goldgof, Peter Mouton, COMPARISON OF STEREOLOGY METHODS FOR ASSESSING AGE-RELATED EFFECTS ON IMMUNOSTAINED BRAIN CELLS, *Innovation in Aging*, Volume 7, Issue Supplement_1, December 2023, Page 687, https://doi.org/10.1093/geroni/igad104.2232

In the originally published version of this manuscript, the author Aidan Anderson was incorrectly listed as Aiden Anderson.

This error has been corrected online.

